# Anthropometric measurements of school-going-girls of the Punjab, Pakistan

**DOI:** 10.1186/s12887-020-02135-4

**Published:** 2020-05-16

**Authors:** Asima Karim, Rizwan Qaisar

**Affiliations:** 1 Department of Basic Medical Sciences, College of Medicine, University of Sharjah, Sharjah, UAE; 2 Department of Physiology & Cell Biology, University of Health Sciences, Lahore, Pakistan

**Keywords:** Anthropometry, Height, Weight, Body mass index, Centile curves, Pakistan

## Abstract

**Background:**

Child’s growth has been considered an important indicator to evaluate health trends in a population and to devise strategies accordingly. The purpose of the present study was to determine most commonly occurring weight abnormalities among school-going girls from Punjab and to compare with international growth references devised by World Health Organization (WHO) and Centre for Disease Control and Prevention (CDC).

**Methods:**

In this cross-sectional study a sample of 10,050 child and adolescent girls from 12 districts, 35 public/private sector schools, located in rural, semi-urban and urban areas of northern, central and southern Punjab were included. Parameters were measured according to standardised techniques and centile curves obtained by Lambda, Mu, Sigma (LMS) method.

**Results:**

The results showed an increase in weight, height and BMI of the Punjabi girls until 15 years. When compared with international growth references, weight and BMI in our population were significantly lowered; however, height was lower during 12–16 years of age and the differences observed were more pronounced with CDC as compared to WHO. When 3rd, 50th and 90th percentiles of weight, height and BMI in our population were compared with international standards, the values were lower in our paediatric population.

**Conclusion:**

The Punjabi schoolgirls significantly differed from CDC and WHO references, and this difference should be taken into consideration for evaluation of growth abnormalities in our paediatric population. However, in the absence of national reference data, WHO standards have been considered more appropriate for comparison.

## Background

The study of growth in children should be viewed at a bigger canvas because this continuous process comes into motion right from the stage of foetus development, to infancy, early childhood and stretches itself to adolescence stage. Somatic growth during childhood and adolescent period is critical because it lays the foundation for the future health status of the individual [1] and linked to adult weight status and cardiovascular health [2]. Monitoring of children’s growth is an important tool to define normative reference values for a population, to assist healthcare providers to identify any abnormality in growth pattern and to devise preventive measures in order to avoid growth abnormalities.

Anthropometry is a widely used, portable, inexpensive, simple and easy to apply technique, which comprises of several body measurements. Anthropometry parameters such as height, weight, body mass index (BMI) are the best tools for evaluation of nutrition status of children and adults [3]. This technique is not only economical and simple but has proven to be close to accuracy and is universally acceptable [4]; being non-invasive method of assessing the growth in the human body. Anthropometric measures reference values are used to monitor long term health values/indicators for describing growth pattern, trends in body growth and development over time, disease risks, nutritional and general health status in children and adolescence [5].

The body mass index (BMI) [body weight (kg) / height (m)^2^] also known as Quetelet Index [6] is though somewhat a crude index for assessing the nutritional parameters, yet is a method of choice for clinical and research purposes. Accurate measurement of height and weight serve as the basis for anthropological attributes to measure excess and under-weight in relation to height of the body. BMI is a very convenient, non-invasive screening tool for the assessment of weight status in children and adolescents for estimating underweight, overweight and obese in the population [7]. Therefore, determining BMI cut-offs and BMI growth curves are useful to track weight trends in a population. Childhood over-weight and obesity is a serious health issue and an independent risk factor for several cardiovascular and metabolic diseases [8]. Previously, obesity was considered the health issue of the developed countries alone but for the last two decades it has become a serious health concern in middle- and low-income countries [9]. Thinness and stunting are also important parameters to assess child’s physical and mental growth. They are associated with higher morbidity and mortality, inadequate intellectual development, poor educational achievement when compared to children with normal growth parameters. Thinness and stunting reflect malnutrition, poor childcare, chronic infections and inappropriate environmental conditions. According to WHO and CDC, BMI-for-age values are recommended to consider underweight in children of school age and adolescents [10, 11].

Pakistan is among those countries which face the dual burden of an increasing prevalence of overweight/obesity and/or thinness/stunting. According to a recent survey from 57 low- and middle-income countries of school-aged children, a dual burden of undernutrition and overnutrition has been reported in Pakistan. However, the incidence of malnutrition leading to stunting and thinness is very high in Pakistani schoolgirls as compared to over-weight/obesity [12]. In the previous studies there is no definite consensus regarding the existence of obesity or thinness in our paediatric population, as pre-existing data suggests either high prevalence of obesity [13], or thinness [12], or both growth abnormalities amongst Pakistani children/adolescents. Moreover, when the weight and BMI-for-age from Pakistani school-aged boys and girls was compared with the WHO references, there was a significant difference [14]. Overweight and obesity estimates were found to be significantly higher and underweight proportion was significantly lower when WHO references were applied to Pakistani children for growth assessment. Data on the growth status of our paediatric female population is extremely scarce in Pakistan. Although healthy growth of the female adolescent population should be an urgent priority as this will determine the health of our future generations, yet girls in our society are victim of gender inequality, violence and other human rights’ abuses. There is a need to vigorously characterise their health status so that better measurements can be taken to reduce the health abnormalities in adolescent female population.

These differences can be attributed to the effect of ethnicity as well as environmental and nutritional status on BMI/ body growth [15–17]. The prevention and control of epidemic of paediatric obesity and/or thinness requires national estimates for overweight, obesity and thinness, as well as an understanding of whether excess weight gain or under-weight exists in our population. Due to changing trends with time in height and weight the observations designed to improve child health depend on the assessment of the body growth being evaluated through updated growth data [18]. In a systematic extensive and large-scaled review the body growth curves generated by WHO were compared with the growth parameters of children from 55 countries representing ethnic groups highlighting the worldwide variation in growth in relation to ethnicity [19]. We have undertaken this study to determine the growth status of adolescent school-going girls in Pakistan and to determine whether international references for estimates of height, weight and BMI-for age can be utilised for growth assessment in our study population.

## Methods

### Study group

The study was conducted by the Department of Physiology & Cell Biology over 24 months starting from January, 2015. Prior approval was obtained from The Institutional Ethical Review Committee of University of Health Sciences Lahore vide number UHS/ERB/22546/2014.

Punjab, with an area of 205,345 Km^2^, is the largest province of Pakistan, having a population of over 110 million [20]. Administratively it has 36 districts, out of which 12 were randomly selected based on their location, topography, climatic conditions and their rural, semi urban and urban status. The study group of 10,050 schoolgirls aged 8–16 years, residing at different cities from Attock in the North to Rahim Yar Khan and Dera Ghazi Khan in the South. The schoolgirls were recruited from 35 different public and private sector schools, located in rural, semi urban and urban areas within the geographical boundaries of the province of Punjab.

**Fig. 1:**
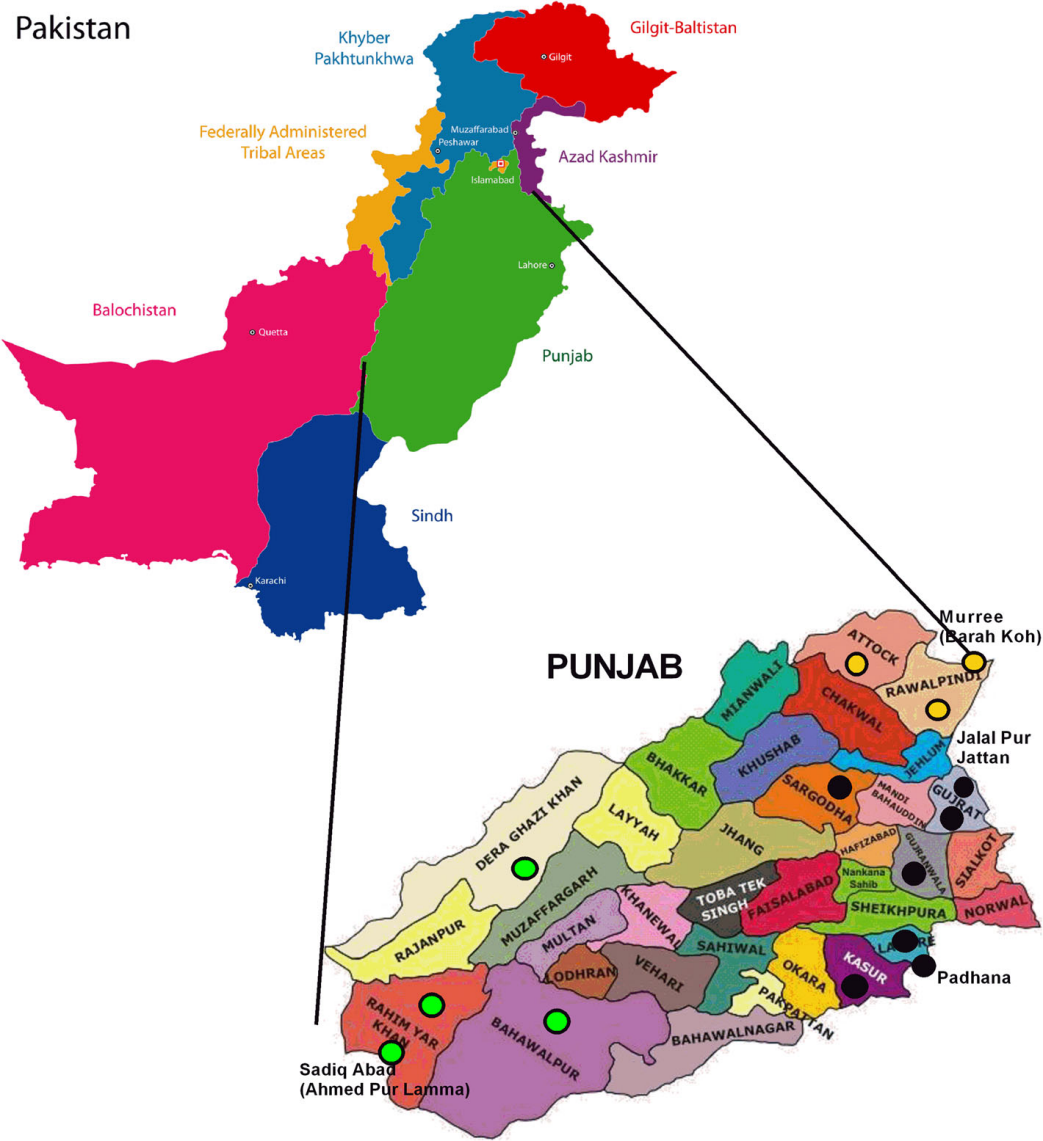
The province of Punjab Pakistan indicating areas visited for collecting the samples. The schools from various areas visited from Central Punjab, Northern Punjab and Southern Punjab shown in black, yellow and green coloured circles respectively (source: Google images, not copyright protected)

In this cross-sectional study stratified multistage cluster sampling technique was employed in different stages. The province of Punjab was considered the stratum and was divided into central, northern and southern Punjab (sub-stratum) keeping in view their geographical location, topography, climatic condition and population for objective sampling. According to the population density, data was collected from 1355 (13.5%) girls from northern Punjab, 6580 (65.47%) from central Punjab and 2115 (21%) from southern Punjab. Twelve out of 36 districts (geographically subdivided by the government) of Punjab and considered as clusters were selected from each identified sub-stratum for data collection (Fig. 1). Districts were stratified using probability proportional to size technique taking into consideration the percentage of adolescent population in each district after obtaining information from Pakistan census bureau [20]. List of all public and private schools of each cluster identified were obtained from the Punjab Department of Education. Stratification of schools was done by considering their rural, semi urban and urban status. Thirty-five public and private schools were randomly selected by Microsoft Excel-generated random numbers. From the finalised schools list the grades were selected based upon the age groups of sample population. The selected schools were contacted through the Department of Education and requested to participate in the study. After obtaining the approval by school administration, list of students in each selected grade were obtained from the school register and students were selected randomly. All the participants were regular and enrolled students of various public/private schools of rural/urban areas of the province. Due consideration and weightage were given to ethics and privacy rights from the beginning. Parents of the girls were informed about the procedure of data collection and prior consents were obtained. Before collecting anthropometric measurements from schoolgirls, basic demographic information was obtained from the school staff and/or parents. Normal and healthy schoolgirls were considered for this study based upon the health records obtained from the respective schools. Girls with any significant past medical or surgical history were not included in this study.

In order to ensure the exact age, the date of birth of each participant was obtained from the school register. The age of the subjects was then calculated from the date of sampling in exact day/month/year. The study population was divided into 12 monthly 9 groups. However, for the analysis and documentation purposes the ages were truncated to full years: thus: for example, 10 years refers to participants aged between 9.5–10.49 years. Moreover, the exact age up to two decimal places was used to generate the percentiles for various anthropometric measurements of the study subjects.

A questionnaire was designed to ask participants relevant questions related to demographic information and previous medical or surgical history relevant to the study. We didn’t measure the impact of socio-economic status on the growth status of our study population. However, study population includes equal representatives of various socio-economic classes therefore, we are confident that socio-economic status has negligible effect on our pooled results. Only few studies have been conducted on representative population from Punjab, however, the data about anthropometric measurements is very inconsistent and warrants the need for a rigorous study covering a large population of school-going girls from various regions of Punjab. We initially conducted a pilot study on 50 school girls from a local school to evaluate the questionnaire, to standardise the measurement protocols, to calibrate equipment according to the prescribed schedule and method, to measure the data with precision and to minimize interference in the school routine activities for evaluating a large number of students. The pilot data obtained clearly revealed that the measurements obtained for height and weight were precise with minimal error, thereby rendering the measurement instruments suitable for data collection. During this study, questionnaire was filled in by authors on their visit to the school one or 2 days prior to the scheduled time for data collection.

### Anthropometric measurements

Anthropometric measurements were recorded for each individual by trained personnel following CDC’s recommendations given in anthropometry procedures manual 2007 by National Health and Nutrition Examination Survey (NHANES) [21]. The assistants in data collection belonged to the medical field and were given extensive training previously in order to ensure the data quality. The anthropometric measurements comprised recording of the weight, height, done in all the subjects.

The same equipment was used to obtain anthropometric data from all the participating subjects for quality assurance. All the measurements were done from eight to eleven in the morning during the school timings with due permission from the Principal/Dean of the participating school.

***Weight*** in Kilograms of the participants was measured with light clothing without shoes using a digital scale (City scale, Fzc, UAE) to the nearest 0.2 Kg. Weighing scale was calibrated with a set of 20 kg weight to ensure accuracy following a standard protocol.

***Standing Height*** is the measurement of maximum vertical size of the body and was measured with a portable Stadiometer (SECA 217, SECA, USA), consisted of a vertical stand and an adjustable headpiece. Height was measured to the nearest of 0.1 cm. Stadiometer was calibrated with a set of predefined lengths to ensure accuracy. While recording the standing height, correct body posture was attained.

#### Statistical analysis

All data entry and analysis were performed using SPSS Version 15.0 (SPSS Inc. Chicago IL, United States, 2009). BMI calculated by the formula BMI = weight (kgs) / height (m^2^). The reference data on L (lambda), M (mu), S (sigma) values and 3rd, 5th, 10th, 15th, 25th, 50th, 75th, 85th, 90th, 95th and 97th percentiles obtained after smoothing for weight, height and BMI by age data of the girls under study by using the software LMS Chartmaker Pro version 2.43 and the centile curves drawn after smoothing the values. Centiles were calculated by LMS and curves generated by the centile values calculated using the following formula: [22]

$$ {C}_{100\alpha }(t)=M\ (t){\left[1+L\ (t)S\ (t)\ {Z}_{\alpha}\right]}^{1/L(t)} $$

where *Z*_*α*_ normal equivalent deviate for tail area *α,* C100*α* weight or height centile corresponding to *Z*_*α*_, *t* age in years, and L(*t*) is skewedness (*t*), M (*t*) is median, S (*t*) is coefficient variation and C_100*α*_ (*t*) shows the corresponding values of each curve at age *t.* The weight, height and BMI, edf (equivalent degrees of freedom) parameters used were L3M5S3, L0M7S4 and L2M4S3, in consultation and under guidance of the support team member of LMS Chartmaker Pro Huiqi Pan, the author of software LMS chartmaker Pro version 2.43 [23] where L stands for Box-Cox power of transformation, M is the median and S is the coefficient of variation, the number after the letter is the edf value. Since weight, height and BMI values changed monotonously with the age therefore, the rescale option used in the LMS chartmaker and the best fitted graphs obtained. The smoothed L values for weight varied between - 1.2 and - 0.3, for height was 1.0 and the value for BMI was between − 0.5 to − 1.5. Data were presented as Mean ± SD for ‘n’ where n representing the number of study participants. The outliers, i.e.*,* values above and below 4SD were considered outliers and not included in the analysis. The outliers were identified from the z-score plot of the parameter under consideration and excluded from the dataset. The mean weight, height and BMI data from the present sample were compared with the weight-, height- and BMI-for-age relative to the WHO (2007) and CDC (2000) references. Statistically significant differences between the means of weight-, height- and BMI-for-age relative to CDC and WHO references, and the present study were calculated with Student’s t-test. One-way analysis of variance (one-way ANOVA) followed by Tukey’s multiple comparison *post-hoc* test was applied for comparison between multiple groups. The level of significance was accepted with *P* < 0.05. The symbols (*and φ) indicated a significant difference between the study population and WHO, CDC references respectively according to Student’s t-test and the hash symbol (#) indicated a significant difference according to ANOVA tests between the groups that were being compared. The single, double and triple symbols exhibited the level of significance for *P* < 0.05, 0.01 and 0.001 respectively.

## Results

A total of 10,050 child and adolescent girls of ages 8 to 16 with a mean of 12.7 ± 2.29 years (Mean ± SD) were evaluated for this study. We measured body weight, height and calculated body mass index (BMI) of this sample population growth centile curves were generated for these parameters and compared the data obtained with the international standards. The results obtained were presented in detail in the following paragraphs.

### Anthropometric measurements

#### Body weight

The data on mean body weight of the subjects (*n* = 10,050) showed a steady increase in the age groups included in the study until the age of 15 years (supplementary Table 1). The first statistically significant increase over the previous age group in mean body weight was observed at the age of 9 years. However no further increase was discernible after 16 years.

**Fig. 2:**
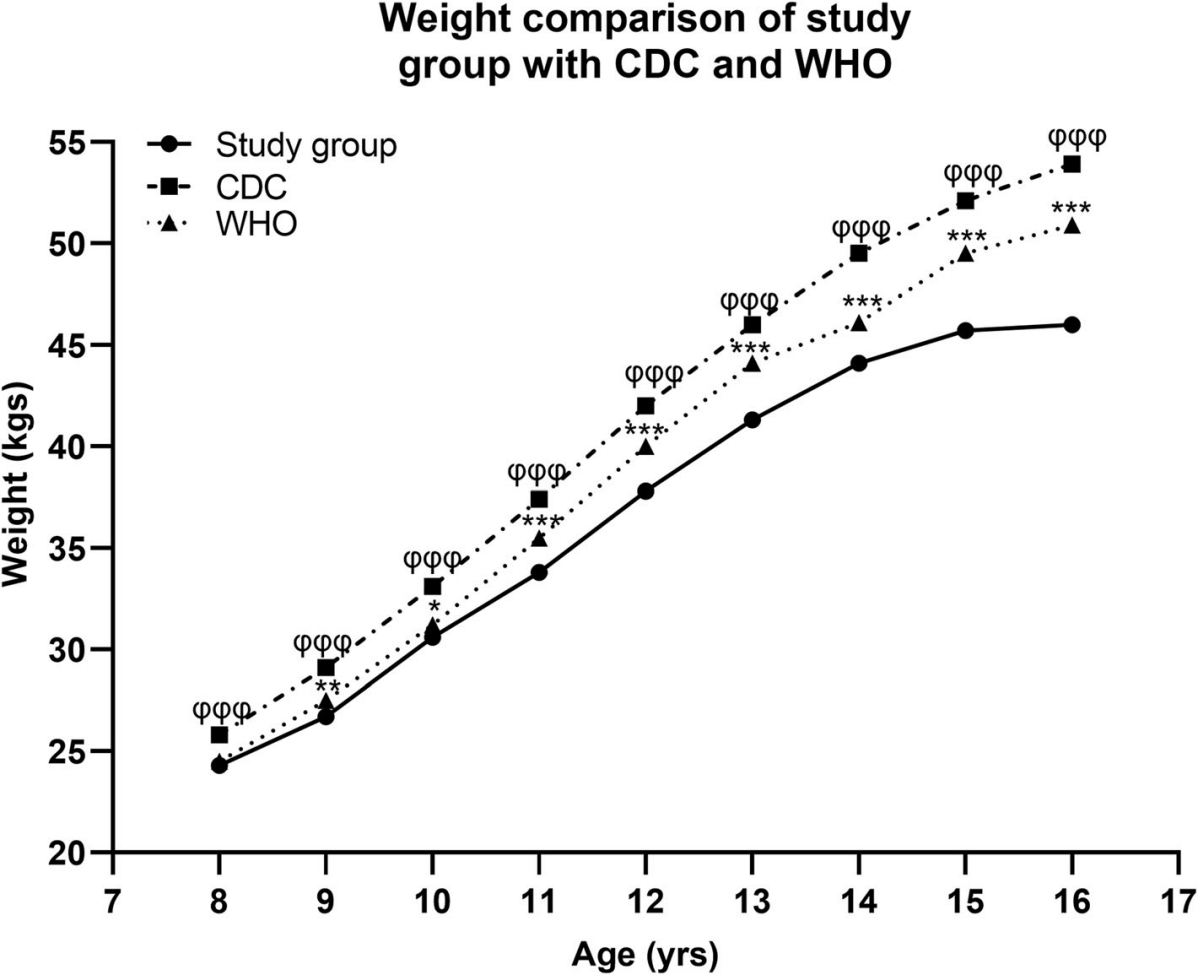
Age related mean BW of study group (solid line) compared with values in the WHO (dotted line) and CDC (broken line) growth reference chart weight-for-age. * and φ showed a significant difference between the mean BW of study group and WHO, CDC references respectively according to Student’s t-test. The single, double and triple symbols exhibited the level of significance for *P* < 0.05, 0.01 and 0.001 respectively

The present data on mean Body weight (Kg) when compared with the CDC [24] and WHO [25] growth references for age matched girls showed an overall trend of lower values of body weight in the study population. The significant difference observed between mean BW of sample and CDC was greater than the difference between sample and WHO standards is shown in Fig. 2 (supplementary Table 2). However, our values were markedly on the lower side especially in the girls of older age groups.

**Fig. 3:**
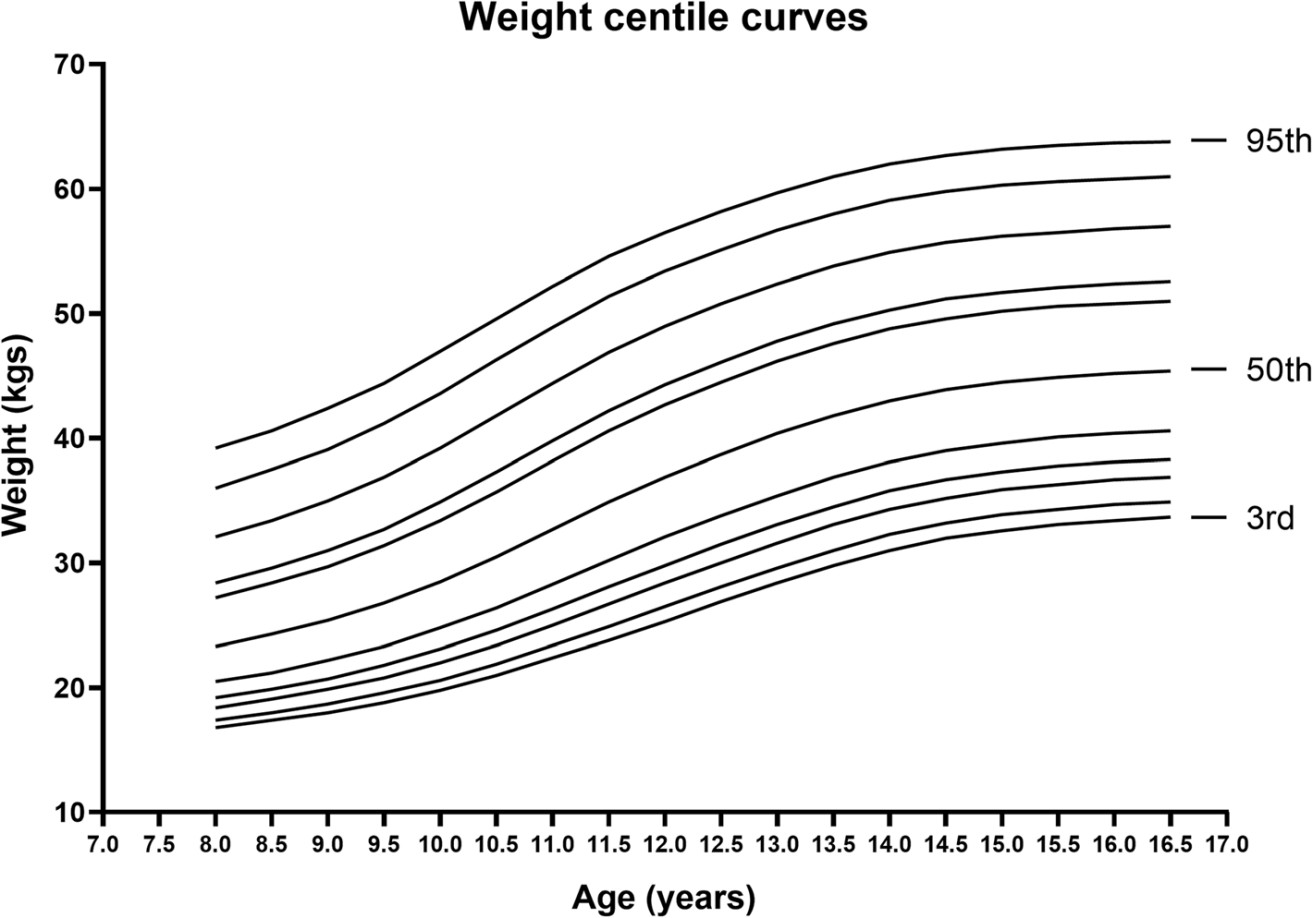
LMS Weight curves for 3rd, 5th, 10th, 15th, 25th, 50th, 75th, 85th, 90th, 95th and 97th percentiles for girls 8–16 years of age

**Fig. 4:**
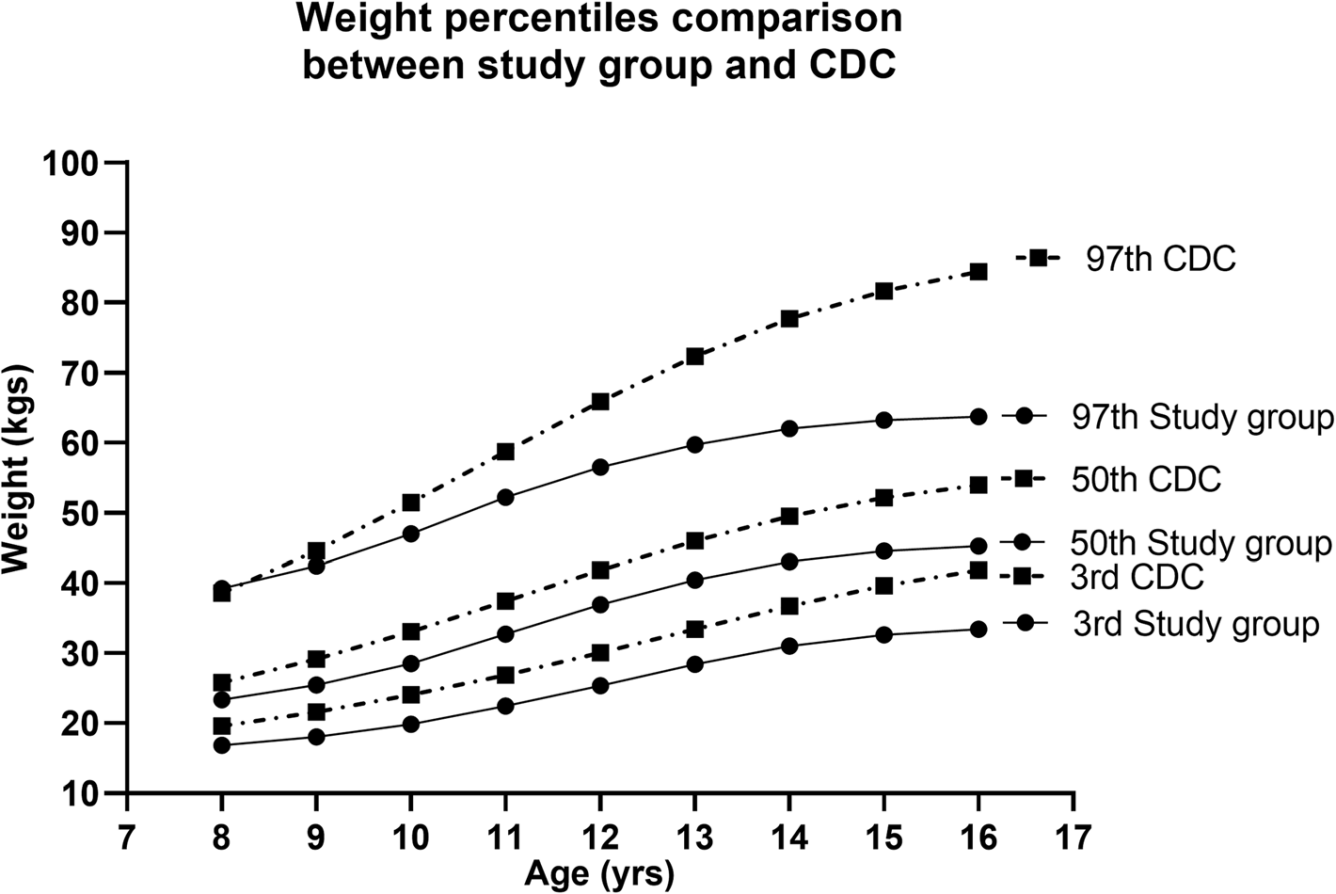
Comparison of selected centiles (3rd, 50th and 97th) of age-related study population weight values (solid line) with the 2000 CDC weight references (dashed line) for Disease Control and Prevention

The reference data on L, M, S values, percentiles obtained after smoothing and the percentile curves generated for weight for age in girls 8–16 years are shown in Fig. 3 (supplementary Table 3). Comparison between the selected percentiles (3rd, 50th and 97th) between the study group and the CDC weight references showed an overall trend of thinness in all the three percentiles in our children shown in Fig. 4 (supplementary Table 4). The values of mean body weight of Punjabi girls in the age range of 8–12 years were found to be comparable with the CDC references on 3rd and 50th percentiles; however, the difference was more pronounced at ages 13–16 years. On the 97th percentile children from 8 to 10 years displayed even lesser difference, but the Punjabi adolescents of 11–16 years had remarkably lower body weight as compared to their CDC counterparts (Fig. 4).

#### Height

Mean height in girls increased during the age groups under study and showed a first statistically significant increase of 4.78 cms at 9 years of age from the previous group. The similar trend existed in all the succeeding ages, with a maximum increase of 5.92 cms seen at the age of 10 years, continued up till 15 years (supplementary Table 5). However, no further increase in mean height observed after 15 years of age.

**Fig. 5:**
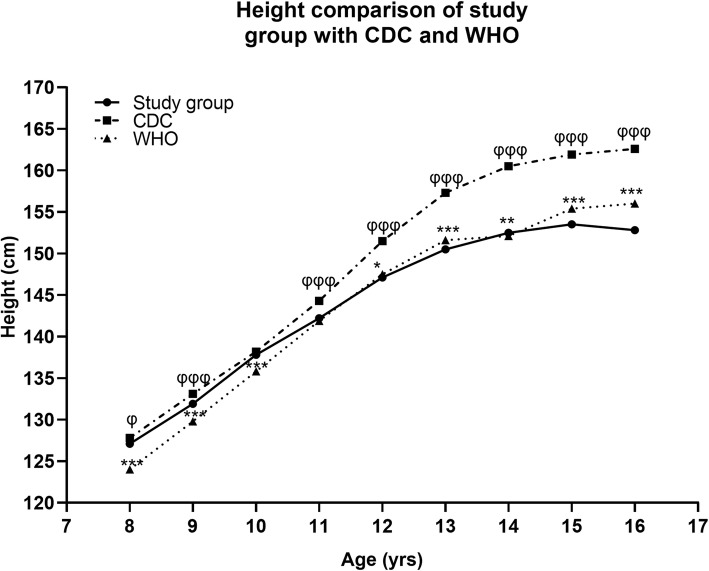
Age related mean height of study group (solid line) compared with values in the WHO (dotted line) and CDC (broken line) growth reference chart weight-for-age. * and φ showed a significant difference between the mean height of study group and WHO, CDC references respectively according to Student’s t-test. The single, double and triple symbols exhibited the level of significance for *P* < 0.05, 0.01 and 0.001 respectively

The present data obtained on mean height (cm) in girls was compared with the international growth references of WHO [25] and CDC [24], trends shown in Fig. 5 (supplementary Table 6). The mean height in our girls had significantly comparable values up to the age of 12 years. However, the height was on the lower side from 12 years of age onwards and the difference was pronounced on comparison with WHO than CDC growth standards.

**Fig. 6:**
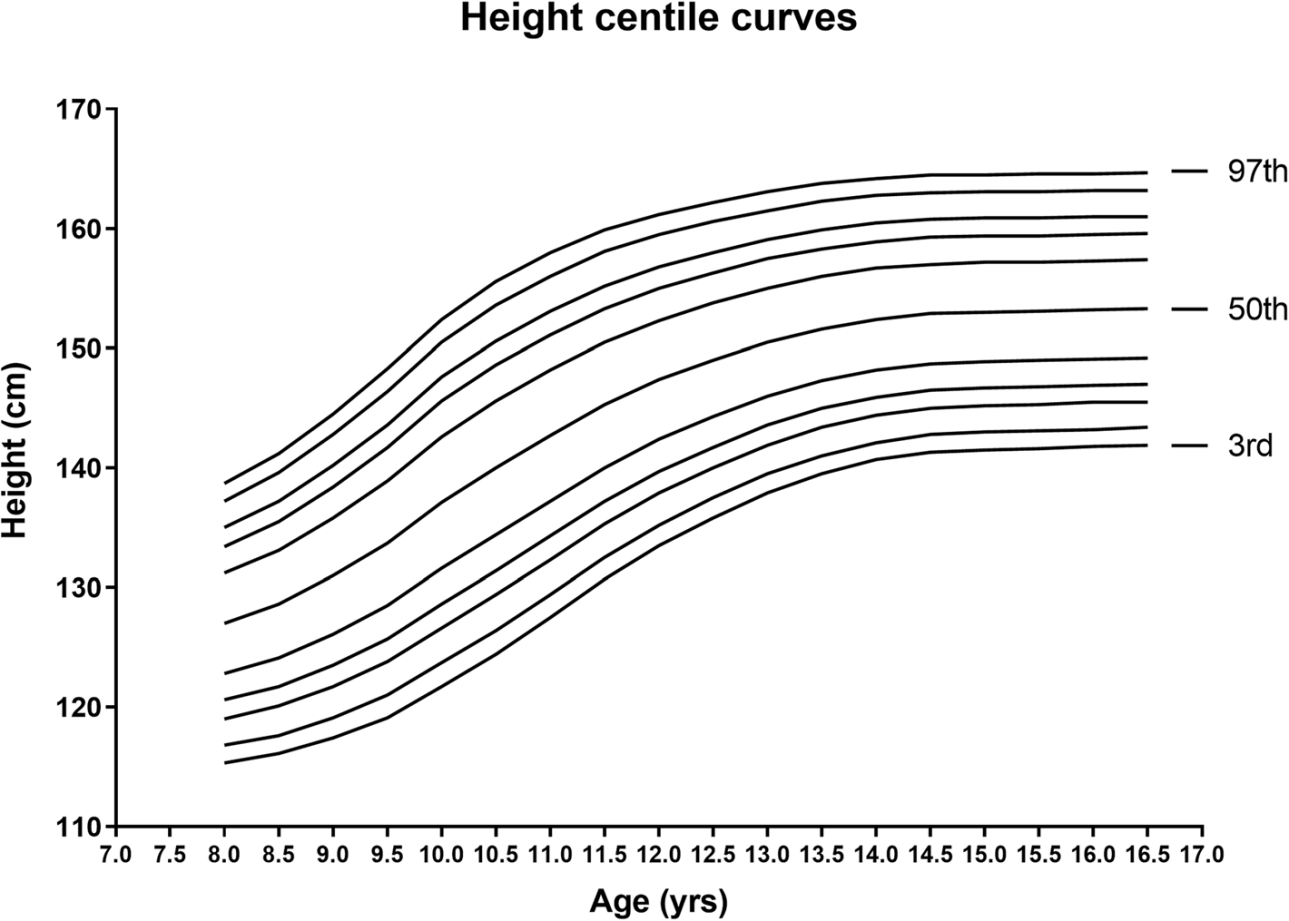
LMS Height curves for 3rd, 5th, 10th, 15th, 25th, 50th, 75th, 85th, 90th, 95th and 97th percentiles for girls 8–16 years of age

**Fig. 7:**
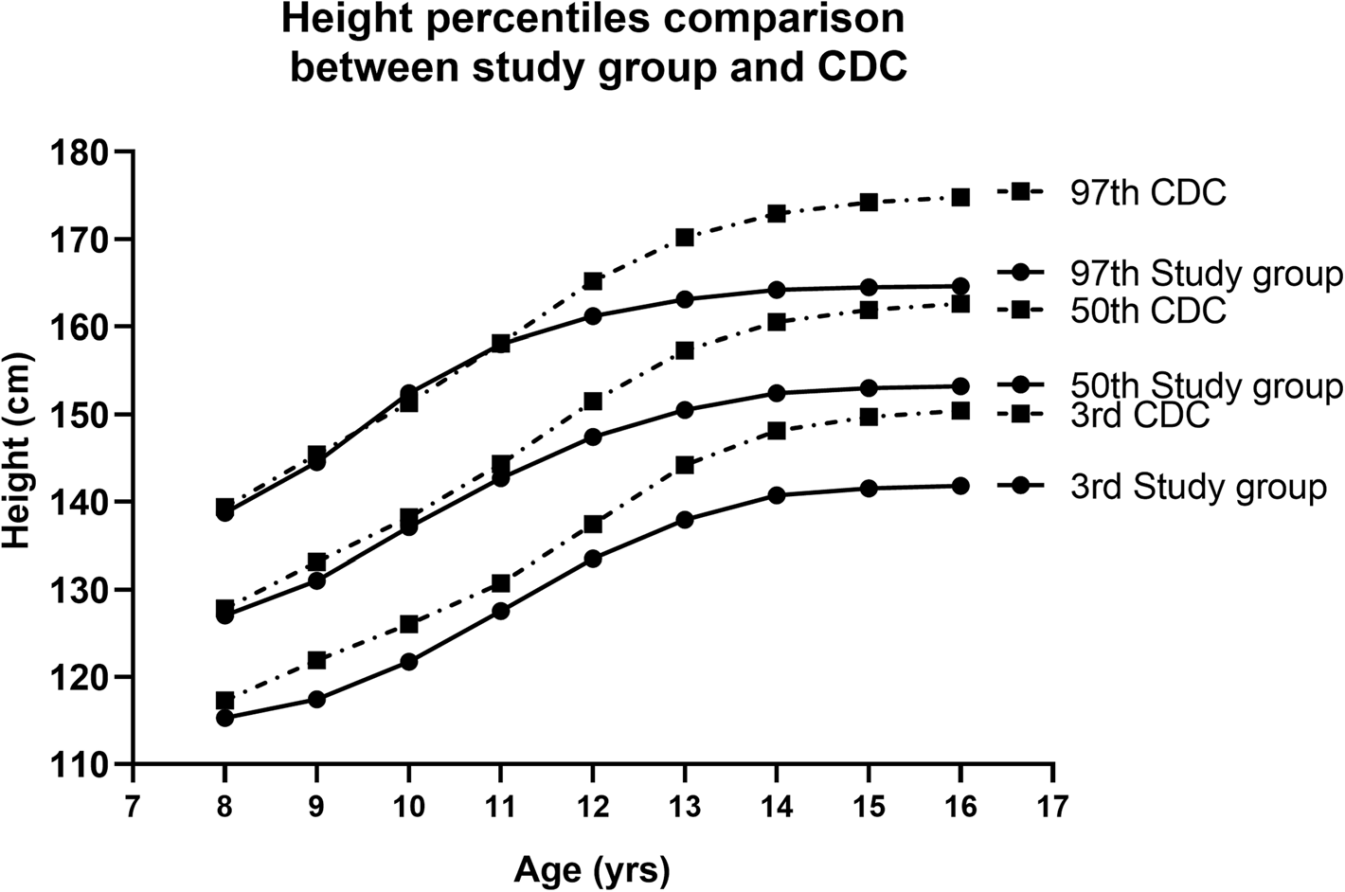
Comparison of selected centiles (3rd, 50th and 97th) of age of study group height values (solid line) with the 2000 CDC height references (dashed line) of the Centres for Disease Control and prevention

The reference values of L, M, S and smoothed percentiles for height by age in girls 8–16 years and the centile curves drawn by Software LMS Chartmaker Pro were displayed in Fig. 6 (supplementary Table 7). Comparison of the selected 3rd, 50th and 97th percentile values of height between the experimental group and the CDC height references revealed that at the 50th and 97th percentiles the height was almost similar between 8 and 11 years of age but the girls in the experimental group were relatively short statured from 12 years of age onwards in these two percentiles shown in Fig. 7 (supplementary Table 8). However, the height values of the 3rd percentile were comparatively on lower side in the girls of all the age groups under study as of their CDC peers.

**Fig. 8:**
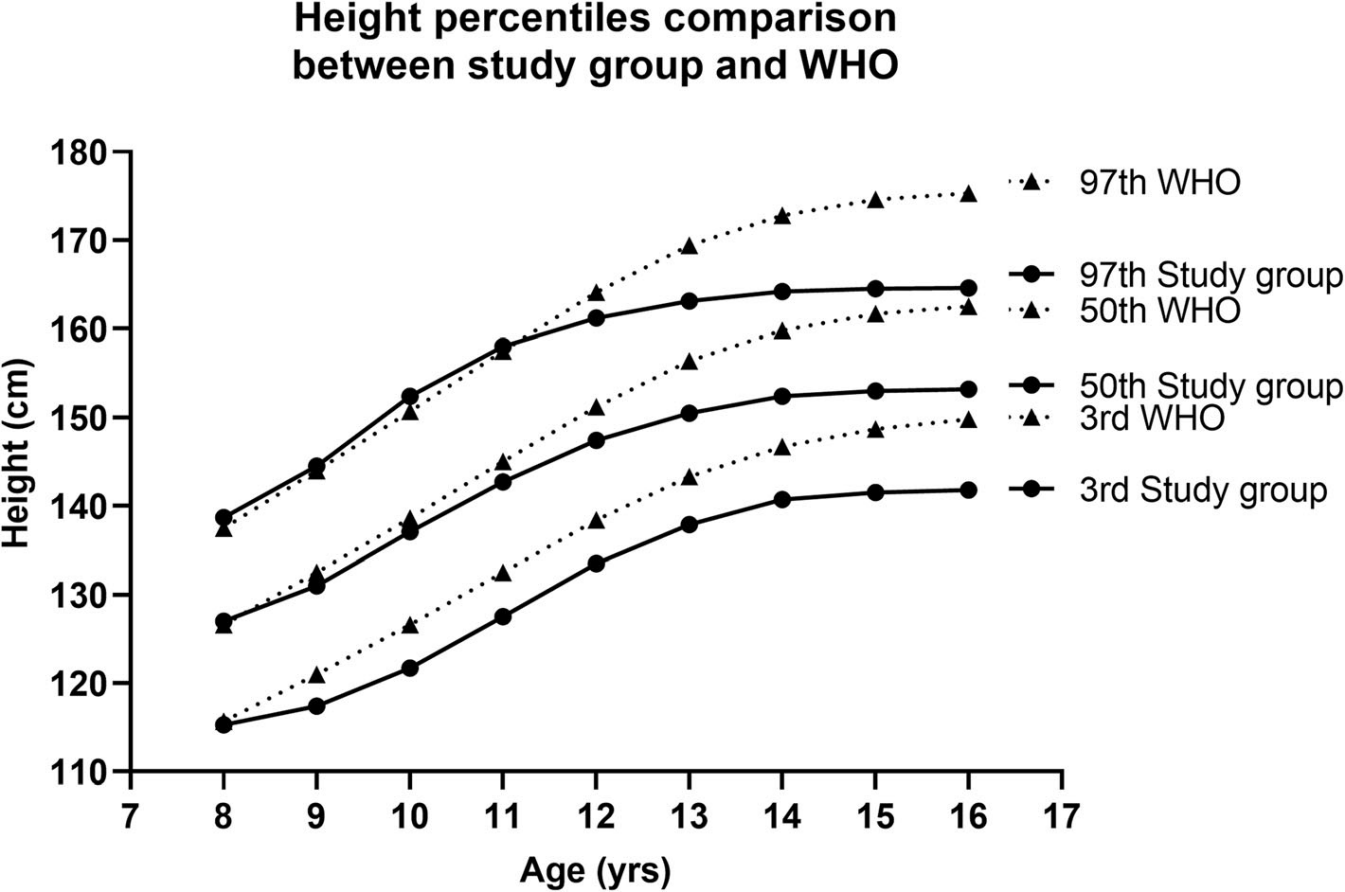
Comparison of selected centiles (3rd, 50th and 97th) of age-related study group height values (solid line) with the WHO height references (dashed line)

A similar trend in the height at the 3rd, 50th and 97th percentiles was observed when the data obtained from the experimental group was compared with the height standards of WHO height for age, graphically represented in Fig. 8 (supplementary Table 9) as that observed on comparison with the CDC standards.

#### Body mass index (BMI)

The mean BMI registered an increase in all the age groups under study with a statistically significant increase from 10 years onward uptil 15 years of age (supplementary Table 10). However, the BMI showed an increase in 16 years of age but statistically non significant.

**Fig. 9:**
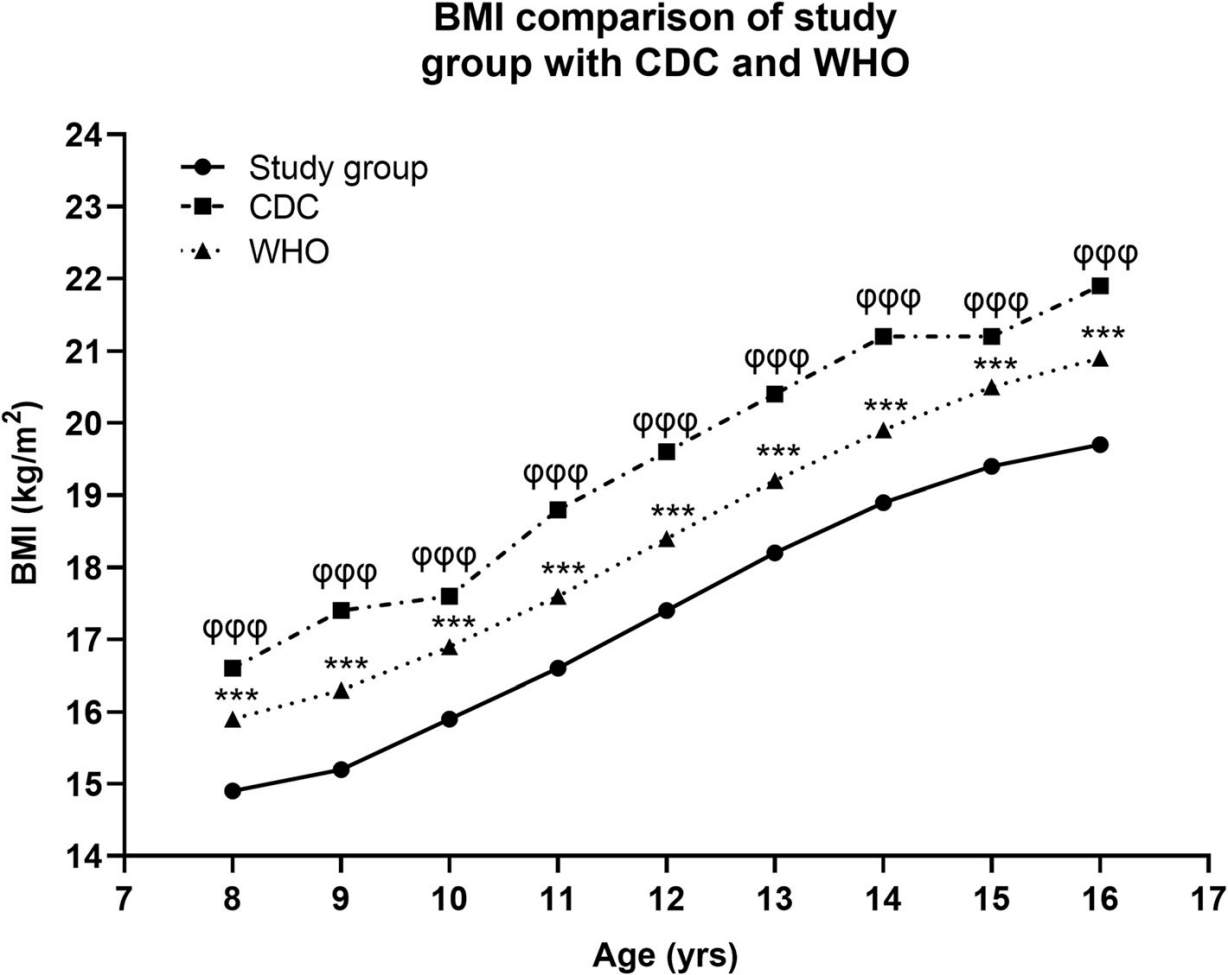
Age related mean BMI of study group of girls (solid line) compared with values in the WHO (dotted line) and CDC (broken line) growth reference chart BMI for-age (24). * and φ showed a significant difference between the mean BMI of study group and WHO, CDC references respectively according to Student’s t-test. The single, double and triple symbols exhibited the level of significance for *P* < 0.05, 0.01 and 0.001 respectively

The age related mean BMI values of the study group girls when plotted in comparison with the international growth standards of WHO and CDC as presented in Fig. 9 (supplementary Table 11), documented significantly comparable data. The mean BMI of the study group was significantly lower in all the age groups (8 to 16 yrs) than the WHO and CDC values, with a more marked diffrence observed on comparison with CDC.

**Fig. 10:**
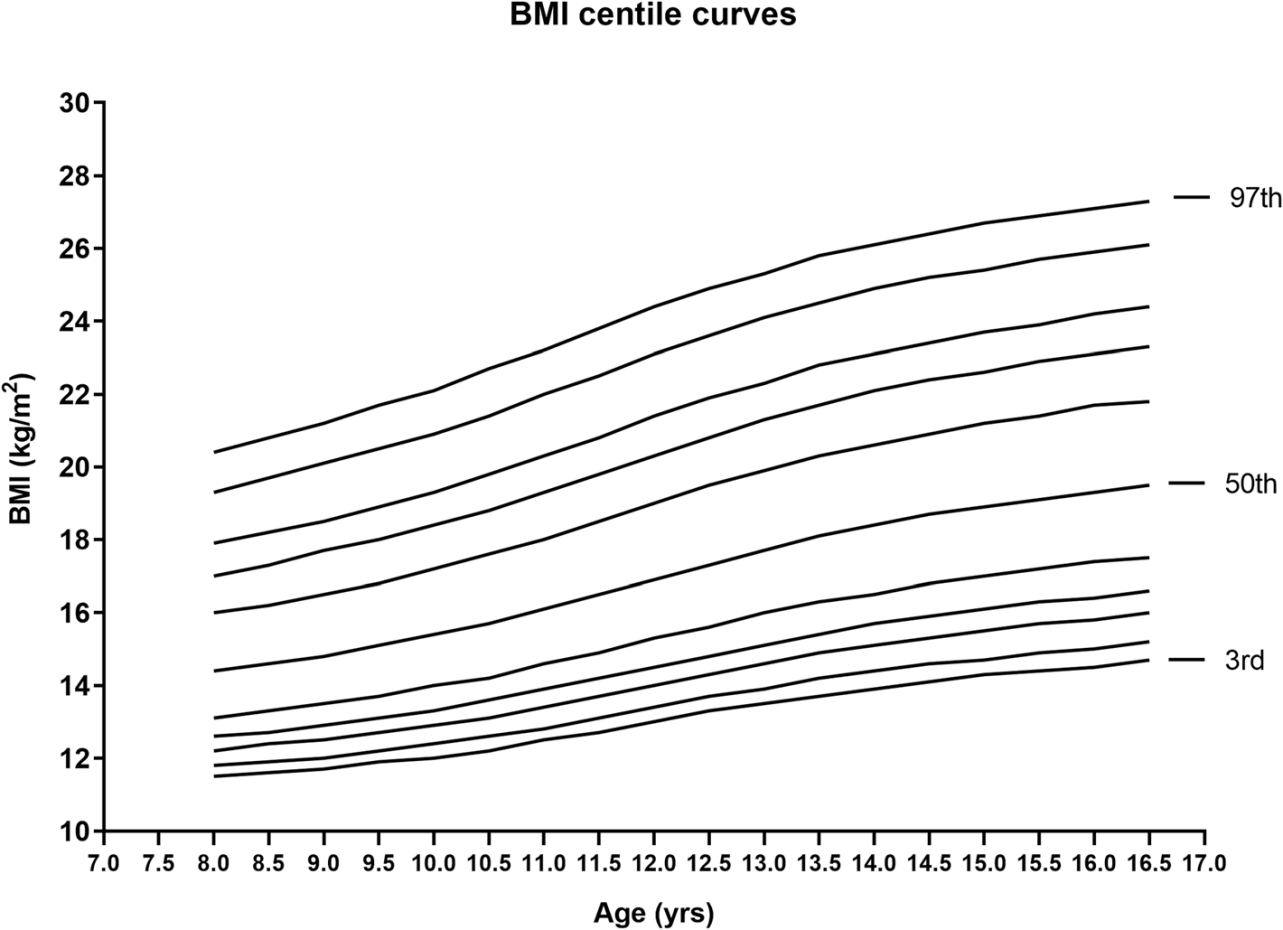
LMS BMI curves for 3rd, 5th, 10th, 15th, 25th, 50th, 75th, 85th, 90th, 95th and 97th percentiles for girls 8–16 years of age

**Fig. 11:**
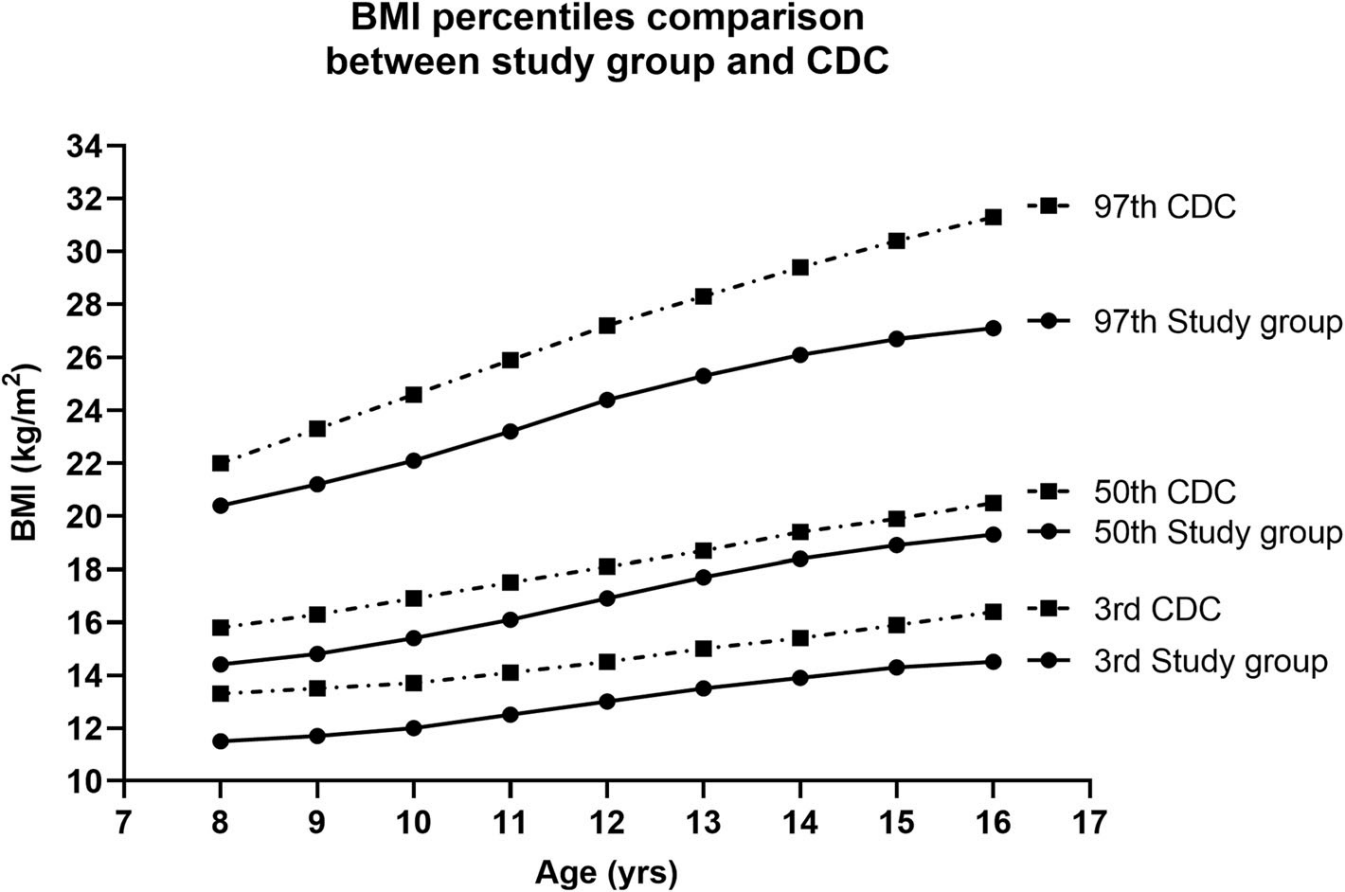
Comparison of selected centiles (3rd, 50th and 97th) of age-related study group BMI values (solid line) with the 2000 CDC BMI references (dashed line) for Disease Control and prevention

The calculated L, M, S values for the reference and the smoothed percentiles obtained from the BMI by age data of the girls under study by using the software LMS Chartmaker Pro version 2.43 and the centile curves drawn after smoothing the values given in Fig. 10 (supplementary Table 12). In a view to compare the BMI percentile curves obtained from the present study with the internationally available percentile curves, 3rd, 50th and 97th percentiles were selected (supplementary Table 13). The present curves obtained were overlapped with the CDC curves in Fig. 11 which clearly displayed an overall trend of thinness in the child and adolescent girls of the study population with a more marked difference seen in the high percentiles. The Punjabi school-going-girls were thinner as compared to their western counterparts in all the age groups under study from 8 to 16 years.

**Fig. 12:**
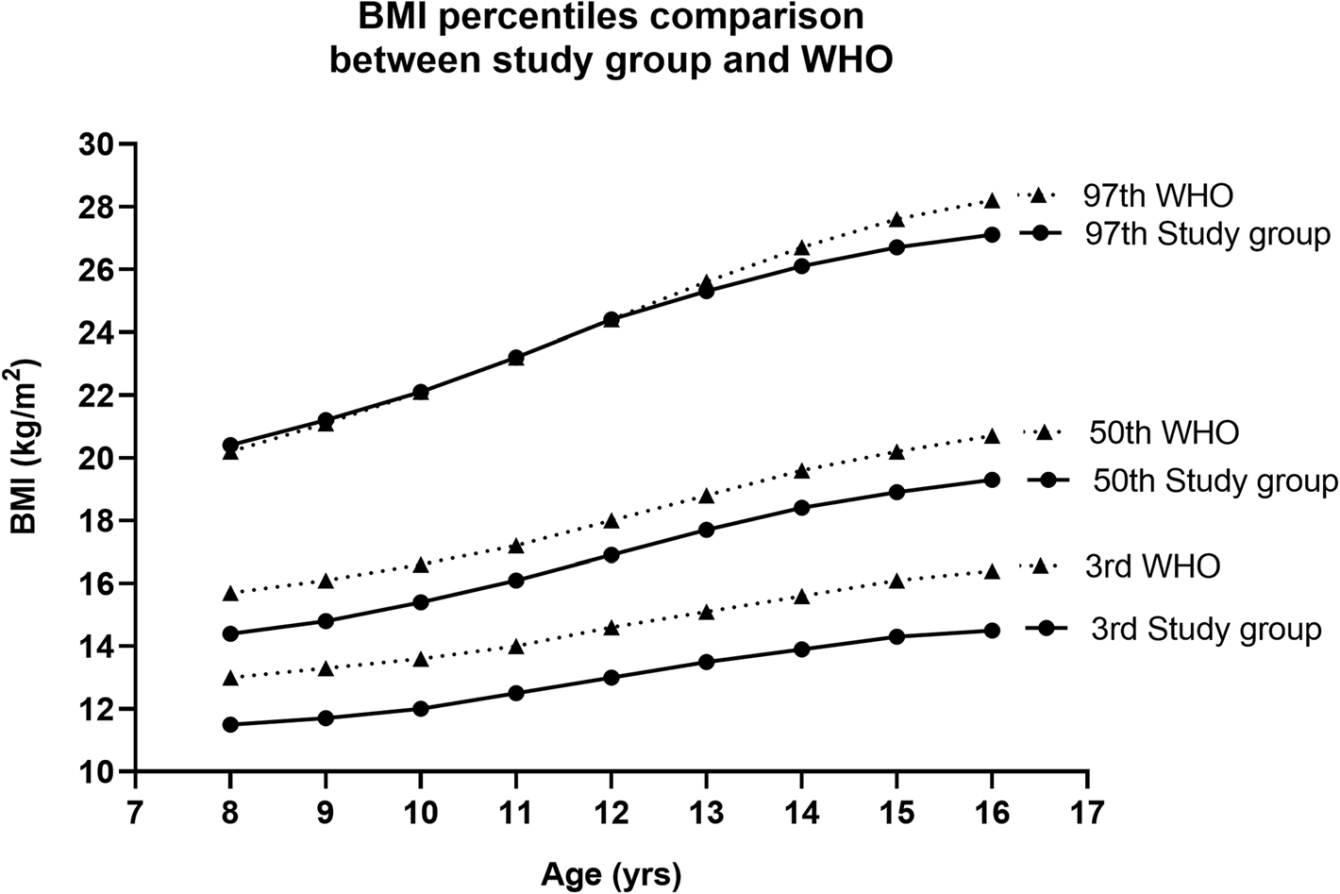
Comparison of selected centiles (3rd, 50th and 97th) of age-related study group BMI values (solid line) with the WHO BMI references (dashed line)

On comparison with the WHO growth standards the values of BMI in the 3rd and 50th percentiles were found to be slightly lower in the girls under study as shown in Fig. 12 (supplementary Table 14). However, an interesting observation was that the BMI in the higher percentiles of the study group was found to be almost like the western population.

## Discussion

Anthropological studies of Pakistani population and especially adolescent girls are practically non-existent. Our population is facing dual burden of child under-nutrition and childhood obesity [26, 27], warranting the need for generating data on growth status of our paediatric population. In the absence of national bench marks, child growth in Pakistani population has been monitored in comparison with international standards and studies have shown that our children differ significantly from WHO and CDC references [14, 28]. Here, we further confirm and extend these findings by reporting reduced height, weight and BMI in school going girls of Punjab, Pakistan when compared to WHO and CDC standards. The lower values persist when data is compared for 3rd, 50th and 90th percentiles of weight, height and BMI. Several previous studies conducted in various countries have shown a significant difference in growth parameters between native and international references, thus emphasising the importance of generating national data [16, 29, 30]. The practical implications of the present study are to determine whether our adolescent girls suffer from obesity or underweight, whether national data is comparable with international standards and which strategies should be adopted to combat with the growth abnormalities in the targeted population.

Weight, height and BMI increased with age in our Punjabi study population till the age of 15 years; however, no further increase in body weight and height was observed at the age of 16 years. When present data was compared with international standards, mean body weight- and BMI-for-age of the Punjabi children and adolescent girls was significantly lower than the WHO and CDC growth references. In our study group, schoolgirls from 8 to 11 years showed comparable values of height-for-age in comparison to CDC and higher values than WHO peers. However, girls from 12 to 16 years of age were lower in height than CDC and WHO counterparts. The difference was more pronounced in comparison with CDC references as compared to WHO references for all the three weight, height and BMI anthropometric measurements. The 3rd, 50th and 97th percentiles of body weight, height and BMI of the study group were compared with CDC and WHO standards. Data revealed that these percentiles in our study group were lower in comparison to the international standards thus indicating the inappropriateness of these standards to be utilised in our population for growth evaluation.

The results of the present study showed that the Punjabi girls were shorter and lighter in comparison to WHO and CDC references. Previous studies conducted in Pakistani children for the assessment of their growth status revealed similar results [14, 28]. The variations observed in this study emphasize the fact that tracing human body growth is a complicated process and several factors can be responsible for this complexity. One possible reason could be the genetic root cause as explained in previous studies [31, 32]. Since the ancestors of most inhabitants of Punjab province have Caucasian roots and migrated from central Asia and eastern Europe [33], the existing differences are less likely to be due to genetic factors alone, though a very high cultural consanguinity of up to ≈80% has been reported in rural and urban population of Punjab in various isonym groups [34, 35]. We are not sure if the variability is due to differences in environment, geographical habitat, culture, socio-economic status and nutrition, since these factors are known to influence growth parameters [36–38]. Moreover, growth monitoring is also affected by secular trends, ethnicity and racial differences [39, 40]. Our study participants were the students of both public and private schools, i.e.*,* belonging to low, low-middle and high-middle socio-economic classes, therefore the differences in body growth found in the present study between local and international growth parameters could be attributed to the economic and indirectly to the nutritional status of paediatric population. We collected data from school going girls alone and not from girls not attending school. However, the latter group, mostly belong to lower socioeconomic status show that their BMI is even lower [41], therefore government needs to take this into consideration when devising health plans. This needs to be characterised for a better estimation of the whole population. Therefore, keeping in mind the nutritional habits and economy of the country, in the present study dietary deficiency may be the possible explanation for the differences in anthropometric measurements of the Punjabi schoolgirls in comparison to the international growth standards.

## Conclusions

In the present study we have generated the centile curves, compared them with international standards (WHO and CDC) and the differences found are noteworthy. The Punjabi schoolgirls of child and adolescent age groups differed significantly from the WHO and CDC references, with comparatively less difference in comparison to WHO rather than CDC growth references.

### Limitations

Firstly, the study was conducted only on girls and the data generated are for the growth profiling of girls, however boys had not been included in this study. Secondly, this study was conducted in the Punjab, one of the provinces of Pakistan. There might be differences in the growth parameters of girls from other provinces, which also needs to be considered in future. Third, the data obtained was from a cross sectional study and shows the growth status of girls at one time point i.e. whether obesity or underweight exist in our population, however this data doesn’t depict the trends in growth, whether obesity or underweight are towards an incline/decline with time. Therefore, longitudinal studies are recommended in future to portray the growth trends in our population at bigger canvas.

## Supplementary information

**Additional file 1 Table 1**: Mean Body Weight ± SD of school-going-girls of the Punjab aged 8–16 years. **Table 2**: Comparison of mean values of weight (Kg) of the study group with CDC and WHO standards. **Table 3**: L, M, S and Percentile values weight (Kg) girls 8–16 years. **Table 4**: Comparison of weight (kg) reference values for school-aged children and adolescent girls for the selected percentiles (3rd, 50th and 97th) of the study group and CDC (24). **Table 5**: Mean Height ± SD of school-going-girls of the Punjab aged 8–16 years. **Table 6**: Comparison of mean values of height (cm) of the study group with CDC and WHO standards. **Table 7**: L, M, S and Percentile values height (cm) girls 8–16 years. **Table 8**: Comparison of height (cm) reference values for school-aged children and adolescent girls for the selected percentiles (3rd, 50th and 97th) of the study group and CDC. **Table 9**: Comparison of height (cm) reference values for school-aged children and adolescent girls for the selected percentiles (3rd, 50th and 97th) of the study group and WHO. **Table 10**: Mean BMI ± SD of school-going-girls of the Punjab aged 8–16 years. **Table 11**: Comparison of mean values of BMI (Kg/m^2^) of the study group with CDC and WHO standards. **Table 12**: L, M, S and Percentile values BMI (Kg/m^2^) girls 8–16 years. **Table 13**: Comparison of BMI (Kg/m^2^) reference values for school-aged children and adolescent girls for the selected percentiles (3rd, 50th and 97th) of the study group and CDC (24). **Table 14**: Comparison of BMI (Kg/m^2^) reference values for school-aged children and adolescent girls for the selected percentiles (3rd, 50th and 97th) of the study group and WHO.

## Data Availability

The data has been included in the form of supplementary tables in this manuscript. The datasets analysed in this study are available from corresponding author upon reasonable request.

## Abbreviations

BMI: Body mass index
CDC: Centers for disease control and prevention
WHO: World health organization
LMS: Lambda, mu, sigma
IREC: Institutional ethical review committee
NHANES: National health and nutrition examination survey
