# Supplementary material for: Anthropometric measurements of school-going-girls of the Punjab, Pakistan
Source: BMC Pediatr. 2020 May 16;20:223. doi: 10.1186/s12887-020-02135-4 (PMC7229613; doi:10.1186/s12887-020-02135-4)
Supplement: Supplementary file 1 — Additional file 1 Table 1: Mean Body Weight ± SD of school-going-girls of the Punjab aged 8–16 years. Table 2: Comparison of mean values of weight (Kg) of the study group with CDC and WHO standards. Table 3: L, M, S and Percentile values weight (Kg) girls 8–16 years. Table 4: Comparison of weight (kg) reference values for school-aged children and adolescent girls for the selected percentiles (3rd, 50th and 97th) of the study group and CDC (24). Table 5: Mean Height ± SD of school-going-girls of the Punjab aged 8–16 years. Table 6: Comparison of mean values of height (cm) of the study group with CDC and WHO standards. Table 7: L, M, S and Percentile values height (cm) girls 8–16 years. Table 8: Comparison of height (cm) reference values for school-aged children and adolescent girls for the selected percentiles (3rd, 50th and 97th) of the study group and CDC. Table 9: Comparison of height (cm) reference values for school-aged children and adolescent girls for the selected percentiles (3rd, 50th and 97th) of the study group and WHO. Table 10: Mean BMI ± SD of school-going-girls of the Punjab aged 8–16 years. Table 11: Comparison of mean values of BMI (Kg/m2) of the study group with CDC and WHO standards. Table 12: L, M, S and Percentile values BMI (Kg/m2) girls 8–16 years. Table 13: Comparison of BMI (Kg/m2) reference values for school-aged children and adolescent girls for the selected percentiles (3rd, 50th and 97th) of the study group and CDC (24). Table 14: Comparison of BMI (Kg/m2) reference values for school-aged children and adolescent girls for the selected percentiles (3rd, 50th and 97th) of the study group and WHO. [file 12887_2020_2135_MOESM1_ESM.docx]

**SUPPLEMENTARY TABLES**

**Table 1**: **Mean Body Weight ± SD of school-going-girls of the Punjab aged 8-16 years**

| **Body Weight (Kg)** | | |
| --- | --- | --- |
| **Age (yrs)** | **Mean** | **SD** |
| 8 | 24.25 | 5.36 |
| 9 | 26.69**^#^** | 6.36 |
| 10 | 30.63**^#^** | 7.61 |
| 11 | 33.84**^#^** | 7.77 |
| 12 | 37.80**^#^** | 6.44 |
| 13 | 41.28**^#^** | 4.36 |
| 14 | 44.10**^#^** | 2.77 |
| 15 | 45.66**^#^** | 1.75 |
| 16 | 45.99 | 1.10 |

Values presented as mean ± SD (n=10,050) and were compared by ANOVA followed by Tukey’s test. *P***^#^** < 0.05 was considered significantly different from the previous age group.

**Table 2: Comparison of mean values of weight (Kg) of the study group with CDC and WHO standards.**

| **Mean Body Weight (Kgs)** | | | |
| --- | --- | --- | --- |
| **Age (yrs)** | **Study group** | **WHO** | **CDC** |
| 8 | 24.3 | 24.5 | 25.8 **^φ φ φ^** |
| 9 | 26.7 | 27.5** | 29.1 **^φ φ φ^** |
| 10 | 30.6 | 31.2* | 33.1 **^φ φ φ^** |
| 11 | 33.8 | 35.5*** | 37.4 **^φ φ φ^** |
| 12 | 37.8 | 40.0*** | 42.0 **^φ φ φ^** |
| 13 | 41.3 | 44.1*** | 46.0 **^φ φ φ^** |
| 14 | 44.1 | 46.1*** | 49.5 **^φ φ φ^** |
| 15 | 45.7 | 49.5*** | 52.1 **^φ φ φ^** |
| 16 | 46.0 | 50.9*** | 53.9 **^φ φ φ^** |

***** and **^φ^** showed a significant difference between the mean BW of study group and WHO, CDC references respectively according to Student’s t-test.

**Table 3:** **L, M, S and Percentile values weight (Kg) girls 8-16 years.**

|  |  |  |  | -1.881 | -1.645 | -1.282 | -1.036 | -0.674 | 0.00 | 0.674 | 0.842 | 1.282 | 1.645 | 1.881 |
| --- | --- | --- | --- | --- | --- | --- | --- | --- | --- | --- | --- | --- | --- | --- |
| **year** | **L** | **M** | **S** | **3rd** | **5th** | **10th** | **15th** | **25th** | **50th** | **75th** | **85th** | **90th** | **95th** | **97th** |
| 8.0 | -1.1115 | 23.3237 | 0.2095 | 16.8 | 17.4 | 18.4 | 19.2 | 20.5 | 23.3 | 27.2 | 28.4 | 32.1 | 36.0 | 39.2 |
| 8.5 | -1.0316 | 24.2768 | 0.2126 | 17.4 | 18.0 | 19.1 | 19.9 | 21.2 | 24.3 | 28.4 | 29.6 | 33.4 | 37.5 | 40.6 |
| 9.0 | -0.9436 | 25.3944 | 0.2158 | 18.0 | 18.7 | 19.9 | 20.7 | 22.2 | 25.4 | 29.7 | 31.0 | 35.0 | 39.1 | 42.4 |
| 9.5 | -0.8438 | 26.7877 | 0.2191 | 18.8 | 19.6 | 20.8 | 21.8 | 23.3 | 26.8 | 31.4 | 32.7 | 36.9 | 41.2 | 44.4 |
| 10.0 | -0.7327 | 28.5312 | 0.2219 | 19.8 | 20.6 | 22.0 | 23.1 | 24.8 | 28.5 | 33.4 | 34.9 | 39.2 | 43.6 | 47.0 |
| 10.5 | -0.6162 | 30.5115 | 0.2232 | 21.0 | 21.9 | 23.4 | 24.6 | 26.4 | 30.5 | 35.7 | 37.3 | 41.8 | 46.3 | 49.6 |
| 11.0 | -0.5014 | 32.6759 | 0.2221 | 22.4 | 23.4 | 25.0 | 26.3 | 28.3 | 32.7 | 38.2 | 39.8 | 44.4 | 48.9 | 52.2 |
| 11.5 | -0.4019 | 34.8555 | 0.2182 | 23.8 | 24.9 | 26.7 | 28.1 | 30.2 | 34.9 | 40.6 | 42.2 | 46.9 | 51.4 | 54.6 |
| 12.0 | -0.3320 | 36.8564 | 0.2120 | 25.3 | 26.5 | 28.4 | 29.8 | 32.1 | 36.9 | 42.7 | 44.3 | 49.0 | 53.4 | 56.5 |
| 12.5 | -0.2985 | 38.6731 | 0.2045 | 26.9 | 28.1 | 30.0 | 31.5 | 33.8 | 38.7 | 44.5 | 46.1 | 50.8 | 55.1 | 58.2 |
| 13.0 | -0.2960 | 40.3506 | 0.1968 | 28.4 | 29.6 | 31.6 | 33.1 | 35.4 | 40.4 | 46.2 | 47.8 | 52.4 | 56.7 | 59.7 |
| 13.5 | -0.3131 | 41.8012 | 0.1895 | 29.8 | 31.0 | 33.1 | 34.5 | 36.9 | 41.8 | 47.6 | 49.2 | 53.8 | 58.0 | 61.0 |
| 14.0 | -0.3378 | 42.9760 | 0.1832 | 31.0 | 32.3 | 34.3 | 35.8 | 38.1 | 43.0 | 48.8 | 50.3 | 54.9 | 59.1 | 62.0 |
| 14.5 | -0.3609 | 43.8510 | 0.1782 | 32.0 | 33.2 | 35.2 | 36.7 | 39.0 | 43.9 | 49.6 | 51.2 | 55.7 | 59.8 | 62.7 |
| 15.0 | -0.3790 | 44.4764 | 0.1746 | 32.6 | 33.9 | 35.9 | 37.3 | 39.6 | 44.5 | 50.2 | 51.7 | 56.2 | 60.3 | 63.2 |
| 15.5 | -0.3920 | 44.9058 | 0.1720 | 33.1 | 34.3 | 36.3 | 37.8 | 40.1 | 44.9 | 50.6 | 52.1 | 56.5 | 60.6 | 63.5 |
| 16.0 | -0.4010 | 45.1960 | 0.1702 | 33.4 | 34.7 | 36.7 | 38.1 | 40.4 | 45.2 | 50.8 | 52.4 | 56.8 | 60.8 | 63.7 |
| 16.5 | -0.4077 | 45.4145 | 0.1689 | 33.7 | 34.9 | 36.9 | 38.3 | 40.6 | 45.4 | 51.0 | 52.6 | 57.0 | 61.0 | 63.8 |

**Table 4:** **Comparison of weight (kg) reference values for school-aged children and adolescent girls for the selected percentiles (3^rd^, 50^th^ and 97^th^) of the study group and CDC (24).**

|  | **3^rd^ Percentile** | | | **50^th^ Percentile** | | | **97^th^ Percentile** | | |
| --- | --- | --- | --- | --- | --- | --- | --- | --- | --- |
| **Age (Y)** | **Study group** | **CDC** | **Diff** | **Study group** | **CDC** | **Diff** | **Study group** | **CDC** | **Diff** |
| 8 | 16.8 | 19.54 | 2.74 | 23.3 | 25.75 | 2.45 | 39.2 | 38.53 | -0.67 |
| 9 | 18 | 21.58 | 3.58 | 25.4 | 29.14 | 3.74 | 42.4 | 44.58 | 2.18 |
| 10 | 19.8 | 23.99 | 4.19 | 28.5 | 33.06 | 4.56 | 47 | 51.42 | 4.42 |
| 11 | 22.4 | 26.81 | 4.41 | 32.7 | 37.39 | 4.69 | 52.2 | 58.72 | 6.52 |
| 12 | 25.3 | 30.01 | 4.71 | 36.9 | 41.82 | 4.92 | 56.5 | 65.9 | 9.4 |
| 13 | 28.4 | 33.4 | 5 | 40.4 | 45.98 | 5.58 | 59.7 | 72.37 | 12.67 |
| 14 | 31 | 36.7 | 5.7 | 43 | 49.49 | 6.49 | 62 | 77.68 | 15.68 |
| 15 | 32.6 | 39.59 | 6.99 | 44.5 | 52.13 | 7.63 | 63.2 | 81.64 | 18.44 |
| 16 | 33.4 | 41.82 | 8.42 | 45.2 | 53.94 | 8.74 | 63.7 | 84.36 | 20.66 |

**Table 5: Mean Height ± SD of school-going-girls of the Punjab aged 8-16 years**

| **Height (cms)** | | |
| --- | --- | --- |
| **Age (yrs)** | **Mean** | **SD** |
| 8 | 127.07 | 6.01 |
| 9 | 131.85 **^#^** | 7.34 |
| 10 | 137.77 **^#^** | 8.18 |
| 11 | 142.16 **^#^** | 7.48 |
| 12 | 147.06 **^#^** | 5.88 |
| 13 | 150.53 **^#^** | 3.53 |
| 14 | 152.54 **^#^** | 2.01 |
| 15 | 153.50 **^#^** | 1.35 |
| 16 | 152.75 | 0.90 |

Values presented as mean ± **SD** (n=10,050) and were compared by ANOVA followed by Tukey’s test. *P***^#^** < 0.05 was considered significantly different from the previous age group.

**Table 6: Comparison of mean values of height (cm) of the study group with CDC and WHO standards.**

| **Mean Height (cm)** | | | |
| --- | --- | --- | --- |
| **Age (yrs)** | **Study group** | **WHO** | **CDC** |
| 8 | 127.1 | 124.0*** | 127.8 **^φ^** |
| 9 | 131.9 | 129.8*** | 133.1 **^φ^** **^φ^** **^φ^** |
| 10 | 137.8 | 135.8*** | 138.2 |
| 11 | 142.2 | 141.9 | 144.3 **^φ^** **^φ^** **^φ^** |
| 12 | 147.1 | 147.5* | 151.5 **^φ^** **^φ^** **^φ^** |
| 13 | 150.5 | 151.6*** | 157.3 **^φ^** **^φ^** **^φ^** |
| 14 | 152.5 | 152.1** | 160.5 **^φ^** **^φ^** **^φ^** |
| 15 | 153.5 | 155.4*** | 161.9 **^φ^** **^φ^** **^φ^** |
| 16 | 152.8 | 156.0*** | 162.6 **^φ^** **^φ^** **^φ^** |

***** and **^φ^** showed a significant difference between the mean height of study group and WHO, CDC references respectively according to Student’s t-test.

**Table 7: L, M, S and Percentile values height (cm) girls 8-16 years**

|  |  |  |  | **1.8808** | **1.6449** | **1.2816** | **-1.0364** | **-0.6745** | **0.0000** | **0.6745** | **1.0364** | **1.2816** | **1.6449** | **1.8808** |
| --- | --- | --- | --- | --- | --- | --- | --- | --- | --- | --- | --- | --- | --- | --- |
| **year** | **L** | **M** | **S** | **3rd** | **5th** | **10th** | **15th** | **25th** | **50th** | **75th** | **85th** | **90th** | **95th** | **97th** |
| **8.0** | **1** | **127.0010** | **0.0489** | **115.3** | **116.8** | **119.0** | **120.6** | **122.8** | **127.0** | **131.2** | **133.4** | **135.0** | **137.2** | **138.7** |
| **8.5** | **1** | **128.6224** | **0.0519** | **116.1** | **117.6** | **120.1** | **121.7** | **124.1** | **128.6** | **133.1** | **135.5** | **137.2** | **139.6** | **141.2** |
| **9.0** | **1** | **130.9723** | **0.0551** | **117.4** | **119.1** | **121.7** | **123.5** | **126.1** | **131.0** | **135.8** | **138.4** | **140.2** | **142.8** | **144.5** |
| **9.5** | **1** | **133.6955** | **0.0579** | **119.1** | **121.0** | **123.8** | **125.7** | **128.5** | **133.7** | **138.9** | **141.7** | **143.6** | **146.4** | **148.3** |
| **10.0** | **1** | **137.0892** | **0.0596** | **121.7** | **123.7** | **126.6** | **128.6** | **131.6** | **137.1** | **142.6** | **145.6** | **147.6** | **150.5** | **152.4** |
| **10.5** | **1** | **139.9818** | **0.0591** | **124.4** | **126.4** | **129.4** | **131.4** | **134.4** | **140.0** | **145.6** | **148.6** | **150.6** | **153.6** | **155.6** |
| **11.0** | **1** | **142.7072** | **0.0568** | **127.5** | **129.4** | **132.3** | **134.3** | **137.2** | **142.7** | **148.2** | **151.1** | **153.1** | **156.0** | **158.0** |
| **11.5** | **1** | **145.2870** | **0.0535** | **130.7** | **132.5** | **135.3** | **137.2** | **140.0** | **145.3** | **150.5** | **153.3** | **155.2** | **158.1** | **159.9** |
| **12.0** | **1** | **147.3705** | **0.0501** | **133.5** | **135.2** | **137.9** | **139.7** | **142.4** | **147.4** | **152.3** | **155.0** | **156.8** | **159.5** | **161.2** |
| **12.5** | **1** | **149.0191** | **0.0471** | **135.8** | **137.5** | **140.0** | **141.7** | **144.3** | **149.0** | **153.8** | **156.3** | **158.0** | **160.6** | **162.2** |
| **13.0** | **1** | **150.5120** | **0.0446** | **137.9** | **139.5** | **141.9** | **143.6** | **146.0** | **150.5** | **155.0** | **157.5** | **159.1** | **161.5** | **163.1** |
| **13.5** | **1** | **151.6475** | **0.0426** | **139.5** | **141.0** | **143.4** | **145.0** | **147.3** | **151.6** | **156.0** | **158.3** | **159.9** | **162.3** | **163.8** |
| **14.0** | **1** | **152.4472** | **0.0411** | **140.7** | **142.1** | **144.4** | **145.9** | **148.2** | **152.4** | **156.7** | **158.9** | **160.5** | **162.8** | **164.2** |
| **14.5** | **1** | **152.8830** | **0.0403** | **141.3** | **142.8** | **145.0** | **146.5** | **148.7** | **152.9** | **157.0** | **159.3** | **160.8** | **163.0** | **164.5** |
| **15.0** | **1** | **153.0298** | **0.0400** | **141.5** | **143.0** | **145.2** | **146.7** | **148.9** | **153.0** | **157.2** | **159.4** | **160.9** | **163.1** | **164.5** |
| **15.5** | **1** | **153.0902** | **0.0398** | **141.6** | **143.1** | **145.3** | **146.8** | **149.0** | **153.1** | **157.2** | **159.4** | **160.9** | **163.1** | **164.6** |
| **16.0** | **1** | **153.2198** | **0.0396** | **141.8** | **143.2** | **145.5** | **146.9** | **149.1** | **153.2** | **157.3** | **159.5** | **161.0** | **163.2** | **164.6** |
| **16.5** | **1** | **153.2896** | **0.0394** | **141.9** | **143.4** | **145.5** | **147.0** | **149.2** | **153.3** | **157.4** | **159.6** | **161.0** | **163.2** | **164.7** |

**Table 8:** **Comparison of height (cm) reference values for school-aged children and adolescent girls for the selected percentiles (3^rd^, 50^th^ and 97^th^) of the study group and CDC.**

|  | | | | **3^rd^ Percentile** | | | **50^th^ Percentile** | | | **97^th^ Percentile** | | |
| --- | --- | --- | --- | --- | --- | --- | --- | --- | --- | --- | --- | --- |
| **Age(Y)** | **Study group** | | **CDC** | | **Diff** | **Study group** | **CDC** | **Diff** | **Study group** | | **CDC** | **Diff** |
| 8 | 115.3 | 117.3 | | | 2.0 | 127 | 127.8 | 0.8 | 138.7 | | 139.4 | 0.7 |
| 9 | 117.4 | 121.9 | | | 4.5 | 131 | 133.1 | 2.1 | 144.5 | | 145.4 | 0.9 |
| 10 | 121.7 | 126.0 | | | 4.3 | 137.1 | 138.2 | 1.1 | 152.4 | | 151.3 | -1.1 |
| 11 | 127.5 | 130.7 | | | 3.2 | 142.7 | 144.3 | 1.6 | 158 | | 158.1 | 0.1 |
| 12 | 133.5 | 137.4 | | | 3.9 | 147.4 | 151.5 | 4.1 | 161.2 | | 165.2 | 4.0 |
| 13 | 137.9 | 144.2 | | | 6.3 | 150.5 | 157.3 | 6.8 | 163.1 | | 170.2 | 7.1 |
| 14 | 140.7 | 148.1 | | | 7.4 | 152.4 | 160.5 | 8.1 | 164.2 | | 172.9 | 8.7 |
| 15 | 141.5 | 149.7 | | | 8.2 | 153 | 161.9 | 8.9 | 164.5 | | 174.2 | 9.7 |
| 16 | 141.8 | 150.4 | | | 8.6 | 153.2 | 162.6 | 9.4 | 164.6 | | 174.8 | 10.2 |

**Table 9: Comparison of height (cm) reference values for school-aged children and adolescent girls for the selected percentiles (3^rd^, 50^th^ and 97^th^) of the study group and WHO.**

|  | **3rd Percentile** | | | **50th Percentile** | | | **97th Percentile** | | |
| --- | --- | --- | --- | --- | --- | --- | --- | --- | --- |
| **Age (Y)** | **Study group** | **WHO** | **Diff** | **Study group** | **WHO** | **Diff** | **Study group** | **WHO** | **Diff** |
| 8 | 115.3 | 115.7 | 0.4 | 127 | 126.6 | -0.4 | 138.7 | 137.5 | -1.2 |
| 9 | 117.4 | 121.0 | 3.6 | 131 | 132.5 | 1.5 | 144.5 | 144.0 | -0.5 |
| 10 | 121.7 | 126.6 | 4.9 | 137.1 | 138.6 | 1.5 | 152.4 | 150.7 | -1.7 |
| 11 | 127.5 | 132.5 | 5.0 | 142.7 | 145.0 | 2.3 | 158 | 157.5 | -0.5 |
| 12 | 133.5 | 138.4 | 4.9 | 147.4 | 151.2 | 3.8 | 161.2 | 164.1 | 2.9 |
| 13 | 137.9 | 143.3 | 5.4 | 150.5 | 156.4 | 5.9 | 163.1 | 169.4 | 6.3 |
| 14 | 140.7 | 146.7 | 6.0 | 152.4 | 159.8 | 7.4 | 164.2 | 172.8 | 8.6 |
| 15 | 141.5 | 148.7 | 7.2 | 153 | 161.7 | 8.7 | 164.5 | 174.6 | 10.1 |
| 16 | 141.8 | 149.8 | 8.0 | 153.2 | 162.5 | 9.3 | 164.6 | 175.3 | 10.7 |

**Table 10**: **Mean BMI ± SD of school-going-girls of the Punjab aged 8-16 years**

| **BMI (Kg/m^2^)** | | | |
| --- | --- | --- | --- |
| **Age (yrs)** | **Mean** | | **SD** |
| 8 | | 14.92 | 2.57 |
| 9 | | 15.20 | 2.45 |
| 10 | | 15.95 **^#^** | 2.82 |
| 11 | | 16.57 **^#^** | 2.88 |
| 12 | | 17.36 **^#^** | 2.24 |
| 13 | | 18.16 **^#^** | 1.66 |
| 14 | | 18.93 **^#^** | 1.13 |
| 15 | | 19.38 **^#^** | 0.71 |
| 16 | | 19.75 | 0.50 |

Values presented as mean ± **SD** (n=10,050) and were compared by ANOVA followed by Tukey’s test. *P***^#^** < 0.05 was considered significantly different from the previous age group.

**Table 11: Comparison of mean values of BMI (Kg/m^2^) of the study group with CDC and WHO standards.**

| **BMI (Kg/m^2^ )** | | | |
| --- | --- | --- | --- |
| **Age (yrs)** | **Study group** | **WHO** | **CDC** |
| 8 | 14.9 | 15.9*** | 16.6 **^φ φ φ^** |
| 9 | 15.2 | 16.3*** | 17.4 **^φ φ φ^** |
| 10 | 15.9 | 16.9*** | 17.6 **^φ φ φ^** |
| 11 | 16.6 | 17.6*** | 18.8 **^φ φ φ^** |
| 12 | 17.4 | 18.4*** | 19.6 **^φ φ φ^** |
| 13 | 18.2 | 19.2*** | 20.4 **^φ φ φ^** |
| 14 | 18.9 | 19.9*** | 21.2 **^φ φ φ^** |
| 15 | 19.4 | 20.5*** | 21.2 **^φ φ φ^** |
| 16 | 19.7 | 20.9*** | 21.9 **^φ φ φ^** |

***** and **^φ^** showed a significant difference between the mean BMI of study group and WHO, CDC references respectively according to Student’s t-test.

**Table 12: L, M, S and Percentile values BMI (Kg/m^2^) girls 8-16 years.**

|  |  | |  |  | -1.8808 | -1.6449 | -1.2816 | -1.0364 | -0.6745 | 0.0000 | 0.6745 | 1.0364 | 1.2816 | 1.6449 | 1.8808 |
| --- | --- | --- | --- | --- | --- | --- | --- | --- | --- | --- | --- | --- | --- | --- | --- |
| **year** | **L** | **M** | | **S** | **3rd** | **5th** | **10th** | **15th** | **25th** | **50th** | **75th** | **85th** | **90th** | **95th** | **97th** |
| 8.0 | -1.5383 | | 14.3861 | 0.1441 | 11.5 | 11.8 | 12.2 | 12.6 | 13.1 | 14.4 | 16.0 | 17.0 | 17.9 | 19.3 | 20.4 |
| 8.5 | -1.4882 | | 14.6027 | 0.1464 | 11.6 | 11.9 | 12.4 | 12.7 | 13.3 | 14.6 | 16.2 | 17.3 | 18.2 | 19.7 | 20.8 |
| 9.0 | -1.4359 | | 14.8330 | 0.1488 | 11.7 | 12.0 | 12.5 | 12.9 | 13.5 | 14.8 | 16.5 | 17.7 | 18.5 | 20.1 | 21.2 |
| 9.5 | -1.3799 | | 15.0894 | 0.1512 | 11.9 | 12.2 | 12.7 | 13.1 | 13.7 | 15.1 | 16.8 | 18.0 | 18.9 | 20.5 | 21.7 |
| 10.0 | -1.3187 | | 15.3830 | 0.1538 | 12.0 | 12.4 | 12.9 | 13.3 | 14.0 | 15.4 | 17.2 | 18.4 | 19.3 | 20.9 | 22.1 |
| 10.5 | -1.2523 | | 15.7180 | 0.1562 | 12.2 | 12.6 | 13.1 | 13.6 | 14.2 | 15.7 | 17.6 | 18.8 | 19.8 | 21.4 | 22.7 |
| 11.0 | -1.1808 | | 16.0980 | 0.1584 | 12.5 | 12.8 | 13.4 | 13.9 | 14.6 | 16.1 | 18.0 | 19.3 | 20.3 | 22.0 | 23.2 |
| 11.5 | -1.1062 | | 16.5081 | 0.1601 | 12.7 | 13.1 | 13.7 | 14.2 | 14.9 | 16.5 | 18.5 | 19.8 | 20.8 | 22.5 | 23.8 |
| 12.0 | -1.0309 | | 16.9237 | 0.1614 | 13.0 | 13.4 | 14.0 | 14.5 | 15.3 | 16.9 | 19.0 | 20.3 | 21.4 | 23.1 | 24.4 |
| 12.5 | -0.9570 | | 17.3304 | 0.1624 | 13.3 | 13.7 | 14.3 | 14.8 | 15.6 | 17.3 | 19.5 | 20.8 | 21.9 | 23.6 | 24.9 |
| 13.0 | -0.8859 | | 17.7185 | 0.1632 | 13.5 | 13.9 | 14.6 | 15.1 | 16.0 | 17.7 | 19.9 | 21.3 | 22.3 | 24.1 | 25.3 |
| 13.5 | -0.8195 | | 18.0738 | 0.1637 | 13.7 | 14.2 | 14.9 | 15.4 | 16.3 | 18.1 | 20.3 | 21.7 | 22.8 | 24.5 | 25.8 |
| 14.0 | -0.7586 | | 18.3902 | 0.1640 | 13.9 | 14.4 | 15.1 | 15.7 | 16.5 | 18.4 | 20.6 | 22.1 | 23.1 | 24.9 | 26.1 |
| 14.5 | -0.7037 | | 18.6661 | 0.1641 | 14.1 | 14.6 | 15.3 | 15.9 | 16.8 | 18.7 | 20.9 | 22.4 | 23.4 | 25.2 | 26.4 |
| 15.0 | -0.6541 | | 18.9076 | 0.1641 | 14.3 | 14.7 | 15.5 | 16.1 | 17.0 | 18.9 | 21.2 | 22.6 | 23.7 | 25.4 | 26.7 |
| 15.5 | -0.6089 | | 19.1215 | 0.1641 | 14.4 | 14.9 | 15.7 | 16.3 | 17.2 | 19.1 | 21.4 | 22.9 | 23.9 | 25.7 | 26.9 |
| 16.0 | -0.5672 | | 19.3149 | 0.1639 | 14.5 | 15.0 | 15.8 | 16.4 | 17.4 | 19.3 | 21.7 | 23.1 | 24.2 | 25.9 | 27.1 |
| 16.5 | -0.5275 | | 19.4964 | 0.1638 | 14.7 | 15.2 | 16.0 | 16.6 | 17.5 | 19.5 | 21.8 | 23.3 | 24.4 | 26.1 | 27.3 |

**Table 13:** **Comparison of BMI (Kg/m^2^) reference values for school-aged children and adolescent girls for the selected percentiles (3^rd^, 50^th^ and 97^th^) of the study group and CDC (24).**

|  | **3^rd^ Percentile** | | | **50^th^ Percentile** | | | **97^th^ Percentile** | | |
| --- | --- | --- | --- | --- | --- | --- | --- | --- | --- |
| **Age (Y)** | **Study group** | **CDC** | **Diff** | **Study group** | **CDC** | **Diff** | **Study group** | **CDC** | **Diff** |
| 8 | 11.5 | 13.3 | 1.8 | 14.4 | 15.8 | 1.4 | 20.4 | 22.0 | 1.6 |
| 9 | 11.7 | 13.5 | 1.8 | 14.8 | 16.3 | 1.5 | 21.2 | 23.3 | 2.1 |
| 10 | 12 | 13.7 | 1.7 | 15.4 | 16.9 | 1.5 | 22.1 | 24.6 | 2.5 |
| 11 | 12.5 | 14.1 | 1.6 | 16.1 | 17.5 | 1.4 | 23.2 | 25.9 | 2.7 |
| 12 | 13 | 14.5 | 1.5 | 16.9 | 18.1 | 1.2 | 24.4 | 27.2 | 2.8 |
| 13 | 13.5 | 15.0 | 1.5 | 17.7 | 18.7 | 1.0 | 25.3 | 28.3 | 3.0 |
| 14 | 13.9 | 15.4 | 1.5 | 18.4 | 19.4 | 1.0 | 26.1 | 29.4 | 3.3 |
| 15 | 14.3 | 15.9 | 1.6 | 18.9 | 19.9 | 1.0 | 26.7 | 30.4 | 3.7 |
| 16 | 14.5 | 16.4 | 1.9 | 19.3 | 20.5 | 1.2 | 27.1 | 31.3 | 4.2 |

**Table 14: Comparison of BMI (Kg/m^2^) reference values for school-aged children and adolescent girls for the selected percentiles (3^rd^, 50^th^ and 97^th^) of the study group and WHO.**

|  | **3rd Percentile** | | | **50th Percentile** | | | **97th Percentile** | | |  |
| --- | --- | --- | --- | --- | --- | --- | --- | --- | --- | --- |
| **Age (Y)** | **Study group** | **WHO** | **Diff** | **Study group** | **WHO** | **Diff** | **Study group** | **WHO** | **Diff** | |
| 8 | 11.5 | 13.0 | 1.5 | 14.4 | 15.7 | 1.3 | 20.4 | 20.2 | -0.2 |  |
| 9 | 11.7 | 13.3 | 1.6 | 14.8 | 16.1 | 1.3 | 21.2 | 21.1 | -0.1 |  |
| 10 | 12 | 13.6 | 1.6 | 15.4 | 16.6 | 1.2 | 22.1 | 22.1 | 0.0 |  |
| 11 | 12.5 | 14.0 | 1.5 | 16.1 | 17.2 | 1.1 | 23.2 | 23.2 | 0.0 |  |
| 12 | 13 | 14.6 | 1.6 | 16.9 | 18.0 | 1.1 | 24.4 | 24.4 | 0.0 |  |
| 13 | 13.5 | 15.1 | 1.6 | 17.7 | 18.8 | 1.1 | 25.3 | 25.6 | 0.3 |  |
| 14 | 13.9 | 15.6 | 1.7 | 18.4 | 19.6 | 1.2 | 26.1 | 26.7 | 0.6 |  |
| 15 | 14.3 | 16.1 | 1.8 | 18.9 | 20.2 | 1.3 | 26.7 | 27.6 | 0.9 |  |
| 16 | 14.5 | 16.4 | 1.9 | 19.3 | 20.7 | 1.4 | 27.1 | 28.2 | 1.1 |  |
